# Utilising Survivor Stories in Domestic Abuse Education for Medical Students

**DOI:** 10.15694/mep.2019.000172.1

**Published:** 2019-09-12

**Authors:** Rebecca Cox, Jane Moore

**Affiliations:** 1 University of Oxford

**Keywords:** Domestic abuse, education, medical students, narrative, patient experience.

## Abstract

This article was migrated. The article was marked as recommended.

Background

1 in 4 women will experience domestic abuse in their lifetime, potentially leading to physical, psychological, and social problems. There has been increasing research showing doctors need to improve identification of domestic abuse and respond appropriately upon disclosure. There is growing evidence for patient-based teaching, using experiences to increase understanding, empathy, and exposure for students.

Methods

We retrospectively compared student feedback on an existing lecture and domestic abuse videos given to 5 ^th^ year medical students in their obstetrics and gynaecology placement, to a format involving the original lecture and videos plus a survivor in person discussing her story.

Results

109 students returned questionnaires. The results showed an improvement overall in 2017 across all questions. In 2017 95.5% of the students strongly agreed that the session improved their understanding of domestic violence, compared to 50% in 2014. In 2017 90.1% of students strongly agreed the session was interesting compared to 48.7% in 2014. Qualitative data recorded positive comments such as “Personal account of DA in the group was really eye-opening, and I feel this really improved my understanding.”

Discussion

Engaging survivors of domestic abuse could help improve teaching and improve understanding in medical students.

## Introduction

In the UK 1 in 4 women will experience domestic abuse in their lifetime. Two women a week are killed by their partner or ex-partner. Domestic abuse is an incident or pattern of incidents of controlling, coercive, threatening, degrading and violent behaviour, including sexual violence ( Women’s Aid, 2018).

Following domestic abuse the impact on the survivor can be significant and long-lasting ( Women’s Aid, 2019), increasing the risk of mental health problems, worsening of chronic illness, substance abuse, chronic pain, and gynaecological problems. When women present to health professionals there is an opportunity for disclosure. However the woman may be afraid that disclosure will worsen her situation, feel ashamed or guilty, and worried about the reaction of the healthcare professional ( DVL London, 2018).

NICE recommends the clinician asks sensitively during routine care about abuse, knows how to respond to disclosure, has knowledge of the safeguarding process, and can disclose to other professionals ( NICE , 2016). There has been increasing research that doctors need better training to identify cases of domestic abuse and respond appropriately when abuse is disclosed ( Ramsey *et al*., 2012, Frank *et al*., 2006). A cross-sectional study of UK medical schools showed there was no standardised domestic abuse teaching and 75% of respondents felt the teaching provided was inadequate ( Potter and Feder, 2017). There is growing evidence for patient-based teaching, using patient experiences to increase understanding, empathy, and clinical exposure ( Lauckner, Doucet and Wells, 2012) for students and doctors. This method of teaching can involve patients talking about their lived experience in audio, video, group work with students, and as a live speaker.

Engaging people who have experienced domestic abuse in teaching sessions is difficult due to the sensitive nature of the topic. For survivors it can be hard to talk about what happened to them and may involve a brave decision to take part. Making and producing videos can be seen as an easier alternative, however may lose the impact of having a person directly in front of the students discussing what they experienced. Instead, a live patient narrative could be a valuable learning experience for students, giving them the chance to experience first-hand the reality of domestic abuse. From a literature search before the project no other projects or studies were found that had used a live patient narrative in domestic abuse teaching.

At the University of Oxford 5 ^th^ year students receive a domestic abuse teaching module, originally designed by a team from Oxfordshire County Council, which included survivor video interviews. In 2017 this was changed to format involving a survivor talking about her experience of domestic abuse.

This project aims to further advance the patient narrative by assessing the impact of the live patient voice.

### Objective

To investigate if a live patient experience narrative improved medical students’ knowledge and understanding of domestic abuse compared to standard lecture/video based teaching from the retrospective analysis of student feedback forms.

## Methods

The participants were all fifth year University of Oxford medical students undertaking their obstetrics and gynaecology rotation, receiving a lecture on domestic abuse at the John Radcliffe Hospital in Oxford. In each year group there are 160 students, divided into 6 cohorts. Data was collected retrospectively from the 2014 and 2017 academic years.

Group A received the original lecture/videos plus the survivor spending one hour describing her personal experience of domestic abuse, discussing her experience of emotional, psychological, and sexual abuse, using her real experiences to explain how domestic abuse can affect an individual. This session was designed to be interactive, encouraging student involvement, the students had twenty minutes at the end of the session to ask questions to the survivor.

Group B received the original lecture on domestic abuse containing information on definitions, risk factors, types, risk assessment, safeguarding, and disclosure, with the same lecturer as the intervention group but without the patient experience component. The standard lecture included three short videos describing psychological abuse and sexual abuse.

Inclusion criteria: 5 ^th^ year medical students attending the domestic abuse lecture and attended the entire lecture session. Exclusion criteria: any student who left early or incompletely filled out feedback form.

Data was collected from medical students’ feedback forms following attending the domestic abuse teaching as part of their obstetrics and gynaecology lectures. This feedback was collected at the end of each lecture session in the form of an anonymous questionnaire. These were obtained from the educational office in the Women’s and Reproductive Health department in the John Radcliffe Hospital.

Data was collected and analysed on Microsoft Excel.

The main outcomes assessed were medical students’ interest and understanding of domestic abuse assessed by anonymous questionnaire.

Questions on survey:

1. I found the content of the workshop interesting?
2. I liked the format of the workshop?
3. I felt that the material in the workshop was pitched at an appropriate level?
4. This workshop has improved my understanding of domestic abuse?

The questionnaire gave a graded response and collected free-text comments, based on a standardised teaching feedback form used in the department.

Ethical approval was not required, as specified by the University of Oxford’s Central University Research Ethics Committee (CUREC) for data collected before the project was formulated, is fully anonymous and not identifiable by researchers (CUREC, 2019).

## Results/Analysis

A total of 109 students returned questionnaires, in each group every student completed a form- a response rate of 100%. 65 students were in group B, and 44 were in group A. Group B included three groups of students receiving the lecture/video session in 2014, compared to group A which had 2 groups receiving lecture/video sessions plus a patient experience session in 2017. There were 20-24 students in each group ( Table 1). None of the students had received prior formal domestic abuse teaching. The lecture was delivered by the same lecturer and the same patient experience tutor each time.

**Table 1.** Table showing questionnaire data collected from students 2014-2017

Question			Rating		
1. content of workshop interesting	strongly disagree	disagree	unsure	agree	strongly agree
May-14	0	0	1	14	5
Jul-14	0	0	2	14	5
Nov-14	0	0	0	2	22
total	0	0	3	30	32
Jul-17	0	0	0	0	21
Sep-17	0	0	0	4	19
total	0	0	0	4	40
2. liked the format of the workshop					
May-14	0	0	1	17	2
Jul-14	0	2	3	15	1
Nov-14	0	0	0	10	14
total	0	2	4	42	17
Jul-17	0	0	0	2	19
Sep-17	0	0	0	4	19
total	0	0	0	6	38
3.material in the workshop pitched at an appropriate level					
May-14	0	0	1	15	5
Jul-14	0	3	2	14	2
Nov-14	0	0	0	7	17
total	0	3	3	36	24
Jul-17	0	0	0	0	21
Sep-17	0	0	1	1	21
total	0	0	1	1	42
4. workshop improved my understanding of issue of domestic abuse					
May-14	0	0	0	14	7
Jul-14	0	2	1	10	8
Nov-14	0	0	0	6	18
total	0	2	1	30	33
Jul-17	0	0	0	0	21
Sep-17	0	0	0	2	21
total	0	0	0	2	42

Results by question:

1. I found the content of the workshop interesting?

**Figure 1.**
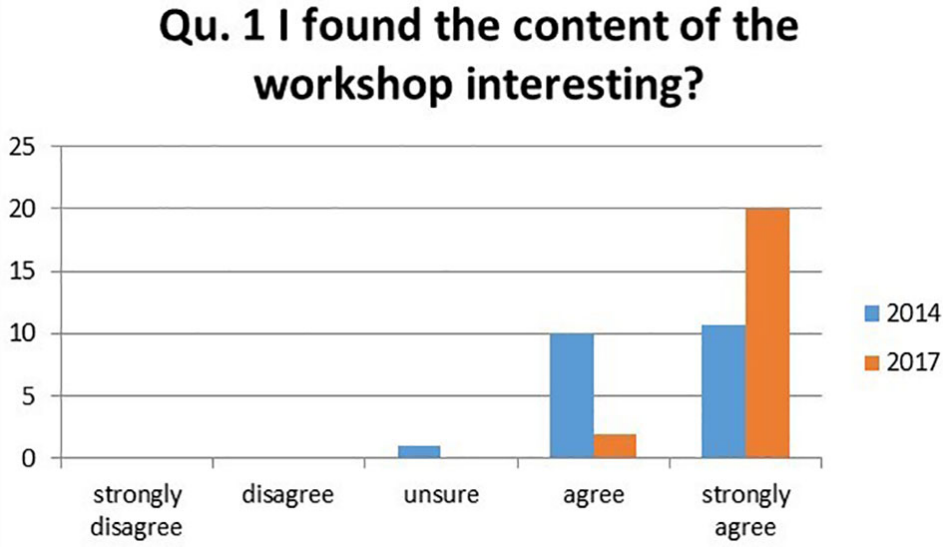
Student’s responses to the question ‘I found the content of the workshop interesting?’

The results showed a positive shift from 2014 to 2017. There was an increase of 41.7% of students strongly agreeing the content was interesting in 2017, with a change from 49.2% students strongly in 2014 to 90.9% in 2017. In 2017 no students rated the content negatively ( Figure 1).

2. I liked the format of the workshop?

**Figure 2.**
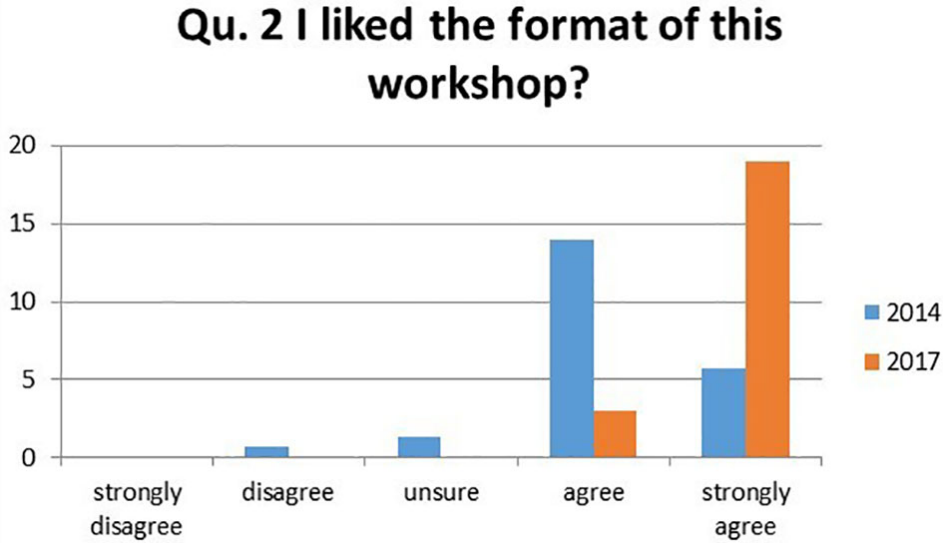
Student’s responses to the question ‘I liked the format of the workshop?’

There was an increase of 60.2% of students strongly agreeing with this statement in 2017, increasing from 26.2% in 2014 to 86.4% in 2017. There is an increased number of students choosing higher ratings in 2017 compared to 2014. There were no negative or unsure rankings in 2017 ( Figure 2).

3. I felt that the material in the workshop was pitched at an appropriate level?

**Figure 3.**
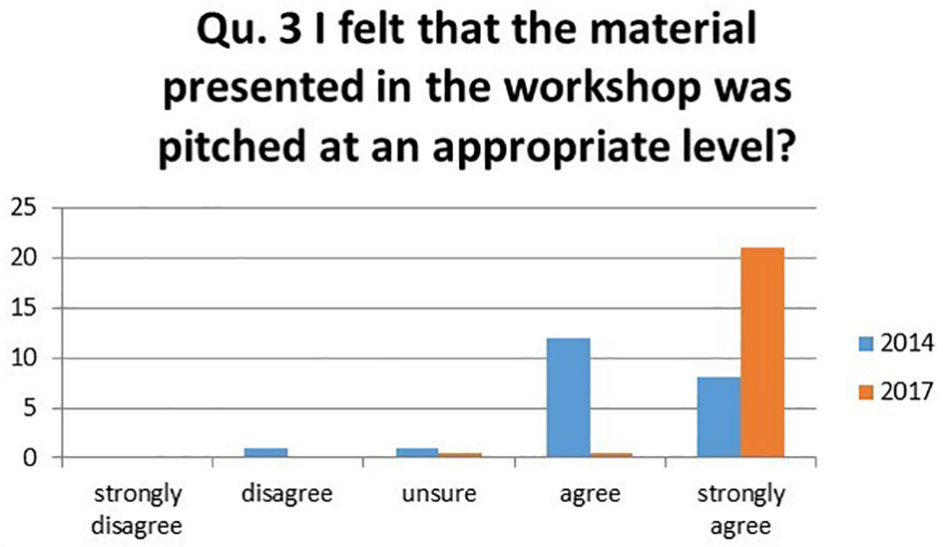
Student’s responses to the question ‘I felt that the material in the workshop was pitched at an appropriate level?’

There was an improvement of 59.1% of students strongly agreeing with the statement in 2017. The majority shifted from selecting “agree” in 2014 to “strongly agree” in 2017. There was a small group of students who chose “unsure” in 2017, but there were no negative rankings ( Figure 3).

4. This workshop has improved my understanding of domestic abuse?

**Figure 4.**
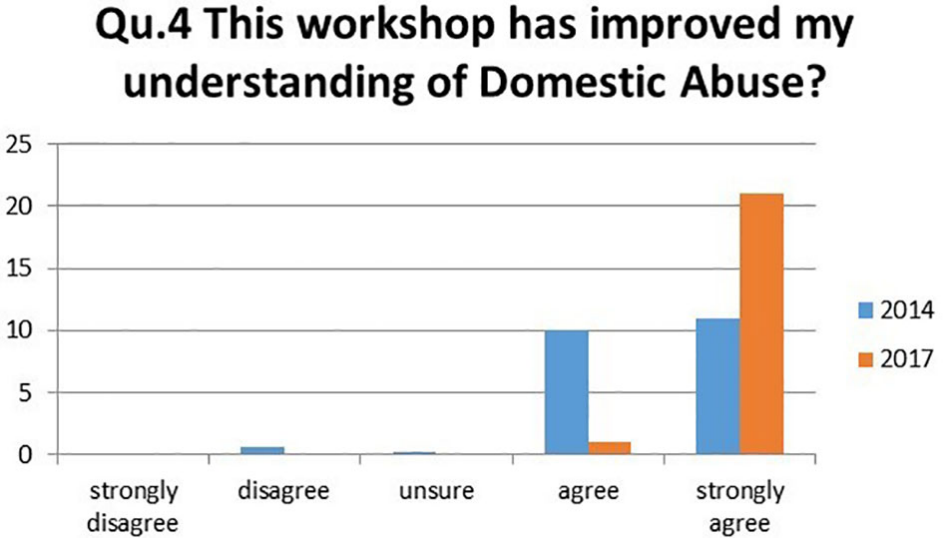
Student’s responses to the question ‘This workshop has improved my understanding of domestic abuse?’

The shift of students strongly agreeing with the statement increased by 45.5% in 2017, growing from 50.0% to 95.5%. This shows an increasing number of students ranking their understanding better in 2017, with all students choosing either “strongly agree” or “agree” ( Figure 4).

Free-text feedback was collected from the questionnaires relating to the patient experience tutor there were 35 positive comments received from the students about this aspect, such as:

“Personal account of DA in the group was really eye-opening, and I feel this really improved my understanding.”

“Learning about the experiences of survivors of domestic abuse was interesting and very emotional - really helped to drive home the importance of the issue”

“Having a speaker with personal experience of abuse, and getting to ask questions”

“First-hand experience was invaluable”

“The personal experience given - helps to understand what to look for. Very very interesting and well presented”

“It was very interesting to hear a personal account of domestic abuse because it makes it more real and memorable, and shows how common it is”

There were no negative comments on the use of the patient experience tutor in the module.

## Discussion

### Main findings

This project found an improvement in all four areas of a questionnaire assessing student feedback on a domestic abuse module for medical students. From the two groups, the students who experienced the live survivor voice found the session more interesting, better format, better content, and most importantly improved their understanding of domestic abuse.

The questionnaire also collected free-text feedback. There were a large number of positive comments on the patient experience component, with no negative comments on this component.

The simple design of this project allowed efficient data collection reviewing questionnaires already completed by students, with every student attending the module completing a questionnaire. The teaching module was repeatedly delivered by the same educators using the same material, at the same time point through the student’s training, reducing variation in the teaching received. Ensuring the students had not received domestic abuse teaching before the module helped to quantify the baseline of their existing knowledge.

The use of a live patient narrative in teaching can be valuable, even more so in topics that heavily involve social problems and lived experiences. Finding a survivor of domestic abuse to talk to a group of students may be difficult due to the nature of the topic, however asking local domestic abuse charities and groups is a great potential resource for teaching.

There is a lack of research evidence using patient experience teaching in domestic abuse education. A large systematic review identified no studies that used live patient experience to educate students or medical professionals. The review found variable responses to online training, physician training with system support, and postgraduate brief interventions ( Zaher, Keogh and Ratnapalan, 2014). A study using a video documentary *Voices of Survivors* containing patient experiences of domestic abuse to educate medical professionals was developed in 2002. A follow-up study showed that the documentary and a session with a domestic abuse advocate improved patient assessment, respect for autonomy, confidence, empathy, knowledge, and self-reported behaviours ( Nicolaidis, Curry and Gerrity, 2005).

### Limitations

Within this project we were limited with student numbers, we sampled 2-3 cohorts from the 6 in each year group. Due to the availability of feedback forms from the lecture groups we had 44 students in group A compared to 65 in group B. The questionnaire provided to students had a limited number of questions, using further questions may give more insight into how the patient experience improved their understanding and knowledge. Furthermore, using a standardised research questionnaire form rather than a departmental pre-designed feedback questionnaire would improve the quality and validity of the data. It is also important to consider the impact of societal changes on the data collected, students may be more aware of gender-based abuse since the increased discussion of women’s rights and health issues.

Future research could involve a formal trial measuring student response to live patient narratives compared to a control on teaching in domestic abuse education, and use qualitative analysis to find themes in written data collected from students.

## Conclusion

This project demonstrates the value of bringing live patient narratives to the emotive topic of domestic abuse. Survivors of domestic abuse could help significantly improve the standards of teaching on this topic and improve understanding in medical students.

Implications for practice

In medical education patient experience can be a valuable educational tool. This method of teaching could enhance and improve domestic abuse teaching in medical schools across the UK.

Implications for research

There is limited research on domestic abuse teaching methods, further research is required on methods of domestic abuse education, finding ways to engage students, and expand outreach and uptake.

## Take Home Messages

• Domestic abuse is a significant health issue affecting mental and physical health.
• Improvement is needed by doctors in recognition of domestic abuse and what to do if a patient discloses.
• Patient narrative teaching is a good method for student learning on topics that are emotive and have a strong patient experience component.
• The live patient voice can increase the learning impact for students compared to video narratives.

## Notes On Contributors

Dr Rebecca Cox: general practice academic clinical fellow at the Nuffield Department Primary Care Health Sciences, University of Oxford, with a special research interest in women’s health, domestic abuse, and pregnancy loss. https://orcid.org/0000-0002-3150-205X

Dr Jane Moore: senior clinical fellow in Nuffield Department of Women’s and Reproductive Health, University of Oxford, and honorary consultant in obstetrics and gynaecology Oxford NHS Foundation Trust.
