# Supplementary material for: Utilising Survivor Stories in Domestic Abuse Education for Medical Students
Source: MedEdPublish (2016). 2019 Sep 12;8:172. [Version 1] doi: 10.15694/mep.2019.000172.1 (PMC10712455; doi:10.15694/mep.2019.000172.1)
Supplement: Peer review history

# Reviewer report: Reviewer response for version 1

**DOI:** 10.21956/mep.19893.r30105

**Published:** 2019-09-26

**Authors:** Suzanne Lady

**Affiliations:** 1 University of Western States

This review has been migrated. The reviewer awarded 5 stars out of 5 It is important for all medical educators to continue to find ways to engage our students and active learning is that way. This paper is valuable in showing that a simple questionnaire given to a group of medical students can show promising teaching techniques that have important outcomes for the learner. In this case, the learner gets first hand experience with a person who experienced domestic abuse. We know from current learning strategies that stories carry the day when it comes to retention and memory. I would like to see a more robust set of survey questions that include confidence levels in these students after having this experience with the live patient. This would help our understanding of how deeply this experience may have impacted the student and what potential future outcome may come from it. The authors acknowledge this in the limitations section.I would recommend this to anyone teaching social dynamics, clinical patient encounters and those deciding what to put unto a curriculum. Great educational topic and very interesting.

Reviewer Expertise:

NA

No decision status is available

# Reviewer report: Reviewer response for version 1

**DOI:** 10.21956/mep.19893.r30104

**Published:** 2019-09-25

**Authors:** Trevor Gibbs

**Affiliations:** 1 AMEE

This review has been migrated. The reviewer awarded 4 stars out of 5 As the world changes, so does our approaches to teaching and learning. I enjoyed reading this paper covering a very important but sensitive subject. The research methodology is simple in its approach , some readers may consider too simple, but I believe it matched its purpose and showed how making any teaching moment more active can affect its learning outcome. I think this approach could be extended to many other examples of teaching and does show the power of patients being involved in teaching and learning.I would recommend this paper to all those involved in curriculum planning and social abuse

Reviewer Expertise:

NA

No decision status is available
